# An Assessment of Climate Change and Health Vulnerability and Adaptation in Dominica

**DOI:** 10.3390/ijerph16010070

**Published:** 2018-12-28

**Authors:** Rebekka Schnitter, Marielle Verret, Peter Berry, Tanya Chung Tiam Fook, Simon Hales, Aparna Lal, Sally Edwards

**Affiliations:** 1 Climate Change and Innovation Bureau, Health Canada, Ottawa, ON K1A 0P8, Canada; Marielle.Verret@Canada.ca (M.V.); Peter.Berry@Canada.ca (P.B.); 2 Department of Geography and Environmental Management, University of Waterloo, Waterloo, ON N2L 3G1, Canada; 3 Faculty of Environmental Studies and Dahdaleh Institute for Global Health Research, York University, Toronto, ON M3J 1P3, Canada; tiamfook@yorku.ca; 4 Department of Public Health, University of Otago, Newtown, Wellington 6242, New Zealand; Haknabus@gmail.com; 5 Research School of Population Health, Australian National University, Acton, Canberra 2600, Australia; Aparnal49@gmail.com; 6 Pan American Health Organization, Washington, DC 20037, USA; Edwardss@paho.org

**Keywords:** Dominica, climate change and health, vulnerability assessment, infectious diseases, food security, severe storm, health system

## Abstract

A climate change and health vulnerability and adaptation assessment was conducted in Dominica, a Caribbean small island developing state located in the Lesser Antilles. The assessment revealed that the country’s population is already experiencing many impacts on health and health systems from climate variability and change. Infectious diseases as well as food and waterborne diseases pose continued threats as climate change may exacerbate the related health risks. Threats to food security were also identified, with particular concern for food production systems. The findings of the assessment included near-term and long-term adaptation options that can inform actions of health sector decision-makers in addressing health vulnerabilities and building resilience to climate change. Key challenges include the need for enhanced financial and human resources to build awareness of key health risks and increase adaptive capacity. Other small island developing states interested in pursuing a vulnerability and adaptation assessment may find this assessment approach, key findings, analysis, and lessons learned useful.

## 1. Introduction

Climate change is a key threat to human health and well-being that requires proactive planning by health authorities [1,2,3]. Improvements to global population health realized over the last number of decades are at risk of being eroded as the climate continues to change [4]. Regional weather and climate changes, including increasing severity and frequency of extreme weather events (EWEs) and changes in temperature and precipitation patterns, can lead to direct and indirect impacts on health and well-being through mediating factors such as social capital, age and gender, health status, demographics, and public health infrastructure. Health effects, including mental illness, malnutrition, allergies, cardiovascular diseases, infectious diseases, injuries, respiratory diseases, and poisoning, arise from direct pathways of climate change effects, such as increasing severity and frequency of storms, floods, droughts and heatwaves, or indirect pathways through changes to water quality, air pollution, land use, and ecosystem changes (e.g., ocean health) [4,5]. These health impacts, particularly to those most vulnerable, can increase pressures on health systems, including damage to infrastructure and disruption of health services, thereby testing the resilience and capacity of the health system to withstand increased shocks and stresses [6,7].

Of the 40 million people that inhabit the Caribbean, over half live within 1.5 km of the coastline, increasing their likelihood of being exposed to extreme weather events, sea-level rise and decreased fisheries productivity [8,9]. People living in small island developing states (SIDS) have been identified as especially vulnerable to the health impacts of climate change [10,11,12,13,14]. These states face common challenges that undermine their resilience to a warming climate, including the existence of small-scale economies, resource scarcity, poor infrastructure, and increased exposure to climate-related hazards, such as EWEs [12,14]. 

The Commonwealth of Dominica is located in the West Indies region of the Caribbean with a population of just over 71,000 people [15]. Climate variability and change are affecting human health in many Caribbean SIDS, such as Dominica, and proactive adaptation planning is required to prepare individuals, communities, and health systems to manage current and future impacts. For example, Dominica has experienced recent outbreaks of climate-related infectious diseases, such as chikungunya and dengue fever (both viral diseases transmitted by the *Aedes aegypti* mosquito), and food production systems in the country have demonstrated sensitivity to gradual climate change as well as climate shocks [16]. In addition, Dominica was struck in August 2015 by Tropical Storm Erika, which caused extensive flooding, landslides, and disruptions to health care in the country [16]. More recently, in September 2017, Dominica was hit by Hurricane Maria, a category five storm, which caused severe destruction across the island. As a result, 31 people died and thousands were injured from landslides and floods. Psychosocial distress was widespread, food security was reduced due to extensive damage to the agricultural sector, and there were significant challenges with accessing safe drinking water [17]. Concerns about vector-borne and waterborne diseases were heightened given the proliferation of vectors and lack of safe drinking water caused by the destruction and accumulated debris on the island [18]. Climate change is making high-intensity storms more likely in the region [19].

Dominica’s 2010–2019 National Strategic Plan for Health acknowledged the health risks of climate change [20], and the country proactively decided to undertake a climate change and health vulnerability and adaptation assessment (V&A), which was completed in 2016 [21]. V&As provide evidence-based information about current and future risks to health, vulnerable populations, and effective adaptation options. They support efforts by public health officials to develop effective measures and build needed partnerships to protect health from climate change impacts [22,23]. The V&A was led by the Dominica Ministry of Health and Environment and received support from the Pan American Health Organization (PAHO), World Health Organization (WHO), World Meteorological Organization (WMO), Health Canada (HC), and the Caribbean Institute for Meteorology and Hydrology (CIMH) [21]. Conducting a V&A is a key action within the PAHO/WHO Strategy and Plan of Action on Climate Change [24], and Dominica was the first country to conduct a national vulnerability and adaptation assessment within the Caribbean region; Grenada completed an assessment shortly after [25].

This paper provides an overview of the V&A conducted in Dominica. It provides information on the assessment approach and the steps followed to investigate climate-related risks to health, populations with higher vulnerability to such risks, and opportunities and challenges with planning for future impacts. Key findings from the assessment are presented along with lessons learned from the study that may be useful to other SIDS undertaking V&As.

## 2. Materials and Methods

The Dominica V&A followed the general methodological approach for undertaking assessments developed by the WHO [21,23]. Due to the limited timeframe and resources, a comprehensive V&A that included all health issues of concern was not undertaken. The steps and related activities that were completed as part of the assessment are presented in Table 1 and are discussed below. Activities that were undertaken in the Dominica study are indicated by a checkmark, and those that were not are indicated with a diamond.

### 2.1. Frame and Scope the Assessment

An advisory committee provided guidance and expertise throughout the assessment process. This committee included members from Dominica’s Ministry of Health and Environment as well as international experts from PAHO/WHO, WMO, HC, and CIMH. The project team also included an international consultant who travelled twice to Dominica to collect data, participate in stakeholder engagement activities, analyze the data, and lead the development of the assessment report. Based on the advisory committee’s expert judgment, four priority health areas were identified for examination in the assessment—vector-borne diseases, waterborne and water-related diseases, foodborne diseases, and food security. Extreme weather events were not initially scoped into the V&A due to time and resource constraints, although they were recognized as an important threat to human health. Tropical Storm Erika struck Dominica while the V&A was being conducted; this resulted in the inclusion of a case study of Erika in the final assessment report.

### 2.2. Conduct the Vulnerability and Adaptation Assessment

A qualitative mixed-methods approach was employed for assessing vulnerability to the health impacts of climate change in Dominica. This approach has been identified as a suitable methodology for SIDS given that quantitative data is often limited or unavailable when conducting assessments [26]. A literature review was conducted to obtain information on climate hazards, exposures, and vulnerabilities relevant to current and future health impacts in the country [27]. Key sources of information for the vulnerability analyses were government departments and stakeholders in Dominica (Table 2). 

To establish baseline conditions, an ecological time series was conducted, modeling the incidence of gastroenteritis in children under 5 years of age and rainfall extremes in Dominica from 1993 to 2008. A distributed lag nonlinear model was used to simultaneously describe nonlinear and delayed dependencies in the association between weekly average rainfall and reported gastroenteritis. Due to resource limitations, it was not possible to model all four of the priority health areas for the assessment.

Stakeholder engagement activities were conducted, including 15 individual key informant interviews, two focus groups, and a national stakeholder workshop that gathered 30 participants. The activities engaged officials from different government ministries, environmental health officers, community leaders, and public health officials. Stakeholder views on climate change risks to health, vulnerable populations, and possible adaptation options were solicited. The qualitative information gathered during stakeholder engagement activities complemented the analysis of the collected quantitative data.

### 2.3. Understand Future Impacts on Health 

Future risks to health from climate change were estimated through a qualitative risk matrix exercise. Stakeholders were provided with climate scenario projections (i.e., temperature and precipitation projections) and asked to estimate the strength of the relationship between climate change in Dominica and each of the four priority health areas as well as assess the severity of the impact.

### 2.4. Identify and Prioritize Adaptation Options

Stakeholders were surveyed about the capacity of individuals, organizations, and the health system in Dominica to respond to increasing risks from climate change. Participants were asked to assess barriers to adaptation and identify existing capacities and opportunities for building resiliency to the health impacts of climate change. Informed by the literature review and the stakeholder engagement activities, an inventory of policy and program adaptation options for enhancing Dominica’s resiliency was developed. Adaptation measures, including opportunities in the near term for enhancing current strategies and measures and longer term actions, were developed for each of the four priority health risk areas under investigation. Analysis of the specific actions was not undertaken (e.g., through a cost-benefit analysis), nor were they prioritized for adoption. While the development of a climate change and health adaptation plan was beyond the scope of this assessment, it is expected that the findings of the study will contribute to the development of a health national adaptation plan (HNAP) by Dominica.

### 2.5. Managing and Monitoring Risks Through an Iterative Process

Due to time and resource constraints, the assessment did not develop a plan that presents an iterative process for managing and monitoring health risks from climate change in Dominica, including measures of health outcomes and the effectiveness of adaptations. As the country moves forward with implementing adaptation measures, it will benefit from developing a plan to regularly assess risks and modify policy and program responses accordingly.

## 3. Results

### 3.1. Vector-Borne Diseases

Dengue, a viral disease transmitted by the *Aedes aegypti* mosquito, represents a priority health risk in Dominica as the disease has a significant epidemic potential [20]. Over the past 25 years, the country has experienced a rise in dengue cases, with significant increases over the last 10 years. As is the case for much of the Caribbean, a seasonal pattern of dengue outbreaks has been observed in Dominica, with the majority of cases occurring between July and December when temperatures are warm and precipitation increases [13,28]. A changing climate may increase transmission rates of vector-borne diseases in Dominica. Increasing temperatures reduce the time taken for virus replication within the mosquito, the extrinsic incubation period. Evidence suggests that increased mosquito survival, reproductive capacity, and biting frequency are also sensitive to temperature [28]. Projected changes in precipitation patterns and increased intensity of hurricane events, combined with inappropriate water storage practices, may lead to an increase in potential mosquito breeding sites [3,29,30,31]. Key informant interviews with health sector experts in Dominica indicated that the strength of the link between vector-borne diseases and climate change and the potential severity of impacts to society is high. 

### 3.2. Food and Water-Related Diseases

Transmission pathways and exposure to food and water-related diseases in Dominica are influenced by climatic, environmental, and socioeconomic factors. Gastroenteritis, which can be transmitted through contaminated food or water sources, is the second most reported syndromic disease in the country [32]. In order to explore the effect of extreme rainfall events on the risk of gastroenteritis, an ecological time-series study of the disease incidence in children under 5 years of age and rainfall extremes was conducted for the period of 1993–2008 in Dominica. 

A significant increase in risk of gastroenteritis was identified within two weeks of extreme dry conditions. Dry periods can lead to a decrease in surface water quality and eventual shortages of stored water. This is of particular concern for communities without reticulated water supplies, where collection of surface water from river and lakes are the main source for drinking, cooking, and sanitation. A marginal increase in the estimated risk of gastroenteritis between 2 and 3 weeks of extreme wet conditions over a 6-week lag was also identified [16]. Although non-climatic factors (e.g., improper solid and liquid waste disposal systems that can lead to contamination of water sources) and specific pathogens were not accounted for, the analysis demonstrates how future studies could use weather variables to identify periods of peak risk for gastroenteritis and inform effective public health interventions.

Leptospirosis is a zoonotic bacterial disease caused by *Leptospira* pathogens. Human cases are most commonly caused by direct or indirect exposure to urine from infected rodents [33]. Indirect exposure often occurs through contaminated water or soil. The rodent population is considered high in Dominica; however, there is no formal rodent surveillance and control program. Poor waste management is considered to significantly contribute to rodent populations in the country [16]. Cases of leptospirosis have increased over the past decade in Dominica, and the disease is considered a significant public health threat [34]. The majority of leptospirosis cases in the country occur during the wet season (December to May), which is consistent with the observed seasonal distribution of the disease throughout the Caribbean region. 

The projected increased intensity of hurricanes, rising temperatures, and changing precipitation patterns from climate change may increase the risk of food and water-related diseases and may compromise access to safe food and water sources in Dominica [13,35,36,37,38]. Key informant interviews with health sector experts indicated that the strength of the link between food and water-related diseases and climate change as well as the severity of impacts to human health is high. 

### 3.3. Food Security

Food insecurity in Dominica is significantly influenced by the health and productivity of the surrounding terrestrial and coastal environments used for food production as well as climate variables, access to food sources, and socioeconomic status. Dominica imports a substantial amount of food [39], and the stability of these imports also influences the population’s exposure to food insecurity. In Dominica, food production systems are highly sensitive to acute and slow onset effects of climate change. The agricultural sector has a high level of exposure and sensitivity to climate risks, including temperature and precipitation variability and extremes, hurricanes, flooding, drought, sea level rise, and soil erosion. Drought, which can lead to decreased plant health and crop losses, is a major concern for the agricultural sector [16]. In 2010, a drought caused losses of 18% of the country’s GDP due to the negative impact on agricultural production [40]. Crop disease and pest outbreaks have already been observed in the country, leading to the decreased quality and productivity of some types of crops [16]. Small-scale coastal fisheries in tropical regions have been identified as particularly vulnerable to the impacts of climate change [41]. Limited studies have been conducted on the health implications of climate change impacts on Dominica’s fisheries sector, although it is an area of increasing concern, especially given the increasing reliance on fisheries production to make up for losses in the agricultural sector. 

For the Caribbean region, projections indicate that islands like Dominica will likely incur agricultural losses of approximately 20% between 2008 and 2050 [42]. Thus, as climate change advances, it is expected that impacts will continue to pose significant threats to food production and, subsequently, food security in Dominica. During key informant interviews, local experts acknowledged a strong link between food insecurity and climate change, recognizing the significant impact to human health. 

### 3.4. Vulnerable Populations

Climate change is a threat multiplier and, among other things, can exacerbate existing health inequities. Population groups that already experience disproportionate vulnerability to social determinants of health, including low-income individuals, the Kalinago community (Dominica’s indigenous population), young children, and the elderly, were identified as experiencing increased vulnerability to climate-change-related health risks. Including considerations of these populations into the development of adaptation initiatives was therefore a primary recommendation in the assessment. The vulnerable groups and key vulnerability factors for each group are summarized in Table 3 by priority health area. 

### 3.5. Adaptive Capacity

Adaptive capacity refers to the ability of a system to adapt to climate variability and change to lessen adverse impacts, build on opportunities, or manage the consequences [43,44]. It includes both current capacity to cope with health threats from climate hazards and future actions that enhance the ability to prepare. Capacity to adapt to climate change risks to health depends upon economic wealth, technology, information and skills, infrastructure, institutions, equity, health status, and pre-existing disease burdens [44]. The V&A gauged adaptive capacity in Dominica through an examination of the following:

- The robustness of Dominica’s health sector
- The adaptive capacity of individuals
- The scope and effectiveness of current adaptations to address priority health risks
- Existing barriers to health adaptation
- Measures Dominica is taking to increase resiliency to climate change health impacts

Stakeholders identified current features of Dominica’s health care system, including the national disease surveillance system and the main health facility in the capital city of Roseau, i.e., Princess Margaret Hospital, as contributing to the country’s resiliency to climate-change-related health risks. However, during the response to Tropical Storm Erika in 2015, the health sector encountered significant challenges in efforts to provide care due to limited road access, damage to health facilities, limited access to water, disruptions to power, limited medical supplies, and reduced staff [16]. In addition, many individuals in the country have low capacity to cope with current climate hazards, including people of low socioeconomic status, indigenous populations (i.e., Kalinago community) and people with low education levels. Food producers were also identified as having lower adaptive capacity given existing environmental and socioeconomic factors that are limiting agricultural productivity in the country [16]. 

A number of measures have been implemented in Dominica to reduce the health risks associated with vector-borne, foodborne, and waterborne diseases and food insecurity. According to stakeholders, the success of vector-borne disease programs has been limited due to low uptake of protective behaviors (e.g., correct water storage in and around homes) among the public. Stakeholders indicated that efforts would benefit from improved waste management practices and enhanced enforcement of related laws and regulations [16]. Programs targeted at reducing waterborne diseases have been more effective given strong stakeholder collaboration and increased awareness among the public regarding safe drinking water practices. In addition, the numerous activities aimed at reducing foodborne diseases have generally been effective, despite the high burden of gastroenteritis in Dominica. 

A number of challenges were perceived to exist in efforts to take action to reduce risks to the health of Dominicans from impacts of current climate variability and to prepare for future climate change. Examples included the following:

- Insufficient training of stakeholders about climate change health risks and adaptation needs
- Insufficient knowledge of health risks from climate change among the public, including a belief by some that climate change is not a real phenomenon
- Lack of financial resources to take needed actions
- Lack of human resources to take needed actions
- Uncertainty about the best adaptation options
- Lack of local data to inform development of adaptation options

In terms of the priority health risks examined in the assessment, vector-borne diseases and food security were identified as having the greatest number of barriers to effective adaptation [16]. Dominica is engaged in a number of climate change resiliency building projects and initiatives at international, regional, and national levels. Table 4 identifies examples of programs, initiatives, and partners that are helping increase the country’s capacity to adapt to the health impacts of climate change.

### 3.6. Adaptation Options to Reduce Health Risks

Irrespective of efforts to mitigate climate change through the reduction of greenhouse gases, adaptation measures are needed to reduce growing risks to health and to protect vulnerable populations [2,3,23]. The assessment identified 92 near-term and longer-term adaptation measures that would help prepare Dominica for climate change by reducing health risks associated with extreme weather events, food insecurity, and vector-borne, waterborne, and foodborne diseases. They include actions to enhance existing risk management activities and additional measures for each priority risk area. Examples are presented in Table 5. 

## 4. Discussion

Small island developing states, such as Dominica, have been identified as being at greater risk from the impacts of climate change, including those on human health and well-being [10,11,12]. Climate models and scenarios suggest future warming of air temperature and increased risk of droughts for Dominica as well as significant warming and sea level rise in the Caribbean [45,46,47]. Severe storms have caused great destruction recently in Dominica, with Hurricane Maria in 2017 and Tropical Storm Erika in 2015. Both of these storms resulted in large numbers of injuries and deaths and significant damage to health systems and services across the country. Climate change is expected to make severe storms more likely, including in the Caribbean [19,46,48].

The climate change and health vulnerability and adaptation assessment in Dominica revealed that the country’s population is already experiencing many impacts on health and on health systems from climate variability and change. With continued changes in temperature and precipitation, infectious diseases remain a key concern given that the island is vulnerable to the re-emergence of malaria [20] as well as the emergence of new arboviral diseases. Dominica recently experienced outbreaks of chikungunya and zika [49] as well as increased cases of dengue [16] and leptospirosis [34] over the last few decades. In addition, foodborne and waterborne diseases that can be exacerbated by climate change pose a continued threat; the number of gastroenteritis cases in Dominica has increased over the last 15 years [16]. 

Given the structure of the Dominican economy and existing environmental and socioeconomic pressures, climate change poses a number of threats to food security. Food production systems are highly sensitive to warming temperatures and extreme events; there has already been a steep rise in plant diseases in a variety of crops and increasing insect infestations. Of particular concern are projections that suggest a reduction in annual crop yields on Caribbean islands, such as Dominica, by 19.65% between 2008 and 2050 [42]. 

A number of populations in Dominica are highly vulnerable to the health impacts of climate change, including low-income individuals, the indigenous Kalinago community, young children, and the elderly. Analysis in the assessment suggested that key vulnerability factors relate to increased physiological sensitivity to hazards (e.g., poor health status), poor housing conditions, reduced access to reticulated drinking water and adequate sanitation, reduced capacity to adapt (e.g., poverty), higher levels of food insecurity, and lower education levels among the population. Importantly, many populations threatened by climate change face multiple health risks, which can make preparedness a greater challenge for individuals, communities, and government officials. 

Opportunities exist for Dominica to build resilience to climate change impacts on health and well-being. A conference and workshop organized in 2003 by WHO to provide information for climate change adaptation planning in the health sector in the Caribbean presented 22 recommendations related to enhancing awareness (e.g., expand knowledge of base relationships between climate and health; promote cross-sectoral communication and consultation in developing adaptations; establish early warning systems), using data (e.g., conduct inventories of existing data; establish better data management systems; identify and map locations, hazards and communities at risk), and strengthening institutions (e.g., evaluate current indicators and regional standards; develop effective mechanisms for information sharing; improve education and training; engage regional and national institutional mechanisms for adaptation development) [11]. In addition, the WHO Operational Framework for Building Climate Resilient Health Systems identifies 10 components related to the building blocks of health systems that constitute a rigorous approach for preparing for the health impacts of climate change. They include actions with respect to the following [50]:

- Leadership and governance
- Health workforce
- Vulnerability, capacity, and adaptation assessment
- Integrated risk monitoring and early warning
- Health and climate research
- Climate resilient and sustainable technologies and infrastructure
- Management of environmental determinants of health
- Climate-informed health programs
- Emergency preparedness and management
- Climate and health financing

Many of the adaptation options highlighted in the Dominica assessment align closely with the recommendations identified by WHO and therefore provide valuable information to health decision-makers and partners in Dominica for moving forward with efforts to plan for climate change and to address the impacts. Preparation for future climate change risks in Dominica would benefit from the expanded collection and analysis of weather, vector, and epidemiologic data and efforts to create a national electronic database for these data that is accessible by relevant stakeholders (Table 5). This information could be used to develop early warning systems for key health risks based upon forecast information provided by regional partners, such as the Caribbean Institute for Meteorology and Hydrology. The effectiveness of such systems would be supported by efforts to enhance the capacity of laboratory facilities to detect disease-causing pathogens and to increase public awareness of health risks associated with existing climate hazards and future climate change. Significant adaptive capacity could be gained by increasing the preparedness and resilience of health facilities in the country. 

Key challenges facing Dominica in efforts to prepare for climate change include the need for enhanced financial and human resources for adaptation to the following [16]:

- Increase awareness among the public and stakeholders about the issue
- Train and educate public health officials and health professionals about growing risks to health from climate change
- Increase local health data that can be linked with climate data including in an electronic format
- Develop knowledge of the most effective adaptation measures
- Build the general capacity of institutions to take action

These requirements are similar to the recommended steps for enhancing climate resilience in Grenada [25]. 

Continued participation in climate-change-related projects and initiatives with key regional and international partners can provide information and expert support toward the development of health adaptation measures in Dominica. With funding from the US National Oceanographic and Atmospheric Administration (NOAA), researchers at several universities, including the University of Arizona, in collaboration with the Caribbean Public Health Agency and local partners, will build on the results of the Dominica V&A for further adaptation planning in that country. They will examine how weather and climate information are currently used in public health decision-making and opportunities to increase climate resilience. Decision support tools will then be developed to facilitate needed actions by public health authorities to protect health from climate-related hazards and prepare for climate change [51]. A monthly Caribbean Health Climatic Bulletin currently provides information to health professionals on favorable or threatening climate conditions in the region so that they may prepare for them [52]. 

Internationally, at the 23rd Conference of the Parties (COP 23) to the United Nations Framework Convention on Climate Change (UNFCCC) in Bonn, Germany, in 2017, the WHO announced a Special Initiative on Climate Change and Health in Small Island Developing States, which has the objectives of empowering health authorities in SIDS to take actions to prepare for climate change, building the evidence to support action, supporting preparedness for climate risks, and facilitating access to financial resources [10]. 

## 5. Conclusions

The climate change and health vulnerability and adaptation assessment in Dominica will serve as a baseline of information about current conditions, projected climate change impacts, and effectiveness of adaptation measures that can be used to chart progress toward building climate-resilient communities and protecting public health. However, the limited scope of the project (e.g., focus on a few key health issues) and the recent impacts from Hurricane Maria mean that further research and capacity building is needed to better understand the breadth of health risks from climate change and the best adaptation options.

## Figures and Tables

**Table 1:** Steps and activities for conducting a climate change vulnerability and adaptation assessment. Source: Reference [23].

WHO Assessment Steps	Dominica Vulnerability and Adaptation Assessment (V&A) Activity
1. Frame and scope the assessment	✓ Define the geographical range and health outcomes of interest ✓ Identify the questions to be addressed and steps to be used ✓ Identify the policy context for the assessment ✓ Establish a project team and a management plan ✓ Establish a stakeholder process ❖ Develop a communications plan
2. Conducting the vulnerability and adaptation assessment	✓ Establish baseline conditions by describing the human health risks of current climate variability and recent climate change and the public health policies and programs to address the risks ✓ Describe current risks of climate-sensitive health outcomes, including the most vulnerable populations and regions ✓ Identify vulnerable populations and regions ❖ Describe risk distribution using spatial mapping ✓ Analyze the relationships between current and past weather/climate conditions and health outcomes ✓ Identify trends in climate-change-related exposures ✓ Take account of interactions between environmental and socioeconomic determinants of health ✓ Describe the current capacity of health and other sectors to manage the risks of climate-sensitive health outcomes
3. Understanding future impacts on health	✓ Estimate future health risks and impacts under climate change ❖ Describe how the risks of climate-sensitive health outcomes, including the most vulnerable populations and regions, may change over coming decades, irrespective of climate change ✓ Estimate the possible additional burden of adverse health outcomes due to climate change
4. Adaptation to climate change: Prioritizing and implementing health protection	✓ Identify and prioritize policies and programs to address current and projected health risks ✓ Identify additional public health and health-care policies and programs to prevent likely future health burdens ✓ Identify resources for implementation and potential barriers to be addressed ❖ Estimate the costs of action and of inaction to protect health ❖ Identify possible actions to reduce the potential health risks of adaptation and greenhouse gas mitigation policies and programs implemented in other sectors ❖ Develop and propose health adaptation plans
5. Establish an iterative process for managing and monitoring the health risks of climate change	❖ Establish an iterative process for managing and monitoring the health risks of climate change

**Table 2:** Key sources of information for the Dominica assessment.

Source	Description
Key data sets (quantitative)	National disease surveillance reports (Health Information Unit) Post-disaster disease surveillance reports (Health Information Unit) National weather and climate data (Dominica Meteorological Service) Demographic data from the national census (Ministry of Finance) Mosquito indices (i.e., household index, Breteau index, container index) (Environmental Health Department) Water quality data (i.e., recreational and drinking) (Environmental Health Department; Dominica Water and Sewerage Company Limited) Environmental health reports on vector control, water quality, and food safety (Environmental Health Department) Food insecurity indicators (Food and Agriculture Organization)
Stakeholder engagement activities (qualitative)	Key informant interviews Focus groups National workshop Surveys Risk matrix exercise
Key stakeholders and partners	Health Centers Health Information Unit, Ministry of Health and the Environment Environmental Health Department, Ministry of Health and the Environment National Pest and Termite Control Dominica Water and Sewerage Company Limited Dominica Meteorological Service Ministry of Agriculture and Fisheries Dominica Organic Agriculture Movement Kalinago People and Territory Dominica Solid Waste Management Corporation Discover Dominica Authority

**Table 3:** Vulnerable groups and associated vulnerability factors by priority health area. Source: References [16,21].

Vector-Borne Diseases	
Vulnerable Groups	Vulnerability Factors
Individuals of low socioeconomic status	Increased reliance on rainwater and/or public standpipes Dominica Water and Sewerage Company Limited (DOWASCO) water disconnections occur more frequently in poorer communities More likely to incorrectly store water in containers Reduced ability to adapt due to financial constraints
Kalinago community	Higher poverty rate than general population, increasing exposure risk Highly dependent on climate-sensitive agriculture and fisheries for food security
Squatters living in “Shanty Towns”	Poor housing conditions leading to increased exposure to vectors
Individuals with poor nutritional status and suffering from micronutrient deficiencies	At higher risk of developing more severe dengue infection
Older persons	Chikungunya can increase mortality in older persons
Infants	Rate of mortality associated with dengue higher in infants
Homebound individuals	Aedes aegypti mosquito known to bite indoors
Farmers	Commonly store water in containers (incorrectly) around farm property, increasing exposure risk
Urban area residents	Higher population density increases exposure risk
Migrants	Increased exposure risk to vectors due to poor housing conditions Reduced access to social and health services
Visitors/tourists	Particularly susceptible to vector-borne diseases
**Food and Water-Related Diseases**	
**Vulnerable Groups**	**Vulnerability Factors**
Those without access to reticulated drinking water	Stored water more likely to become contaminated during heavy rainfall
Individuals without access to improved sanitation	Lack of improved sanitation known to increase risk of transmission
Those living in areas vulnerable to landslides and flooding	Higher risk of water contamination during these events
Those living near agricultural lands	More vulnerable to chemical contamination of water
Migrants living in “Shanty Towns”	Poor housing quality and water management increase exposure risk; reduced adaptive capacity
Recreational water users	Recreational water quality testing indicates sources often do not meet standards
Farmers and fisher folk	Increased exposure risk
Certain occupational groups (e.g., abattoir workers and veterinarians)	Increased exposure risk for abattoir workers, meat handlers, and veterinarians
Infants and children aged <5 years old	This age group is disproportionately affected by gastroenteritis Increased sensitivity
Malnourished children	More sensitive to gastroenteritis, and infection can worsen state of malnourishment
Older persons	Sensitivities related to medications they may be taking, immune response, and physiology
Individuals of low socioeconomic status	May be less educated about safe food and water practices Lack of access to safe drinking water and improved sanitation Limited adaptive capacity
Females	Increased exposure risk (e.g., food preparation) and possible biological factors
Pregnant women and their newborn children	Infection may lead to low birth weight
Immunocompromised individuals	More sensitive to gastroenteritis as infection can become debilitating and life threatening
Persons who eat out regularly	Higher risk of being exposed to parasitic organisms
Visitors/tourists	Unaccustomed to being vigilant when purchasing food and water
**Food Insecurity**	
**Vulnerable Groups**	**Vulnerability Factors**
Individuals of low socioeconomic status and their dependents	Limited purchasing power and more vulnerable to food price increases
Kalinago community	High poverty levels and already established low food security levels Younger generation largely uninterested in farming and fishing
Small-scale farmers and fishers	Depend on agricultural productivity and fisheries catches for stable sources of food and their livelihoods

**Table 4:** Examples of programs, initiatives, and partners that increase Dominica’s adaptive capacity. Source: Reference [16].

**National Programs and Initiatives**
Public health surveillance Water quality monitoring Food Handlers Certification Program Inspection of food establishments Inspection of food at ports of entry Community clean-up campaigns Island-wide solid waste collection and management Health messaging during critical periods Health promotion through radio and television channels Household inspections for mosquito breeding sites Chemical and biological treatments to control mosquito populations Agro-Meteorological Bulletin issued by the Dominica Meteorological Service
**Regional Programs, Initiatives, and Partners**
Smart Hospital Initiative (PAHO/WHO) Caribbean Disaster Emergency Management Agency The Caribbean Public Health Agency Caribbean Regional Fisheries Mechanism Caribbean Coral Reef Watch issued by the Caribbean Regional Climate Centre Climate Change Adaptation in the Eastern Caribbean Fisheries Sector Global Environment Facility (World Bank)—Pilot Program for Climate Resilience
**International Programs and Initiatives**
Global Framework for Climate Services German Agency for International Cooperation (GIZ) programs (e.g., Caribbean Aqua-Terrestrial Solutions) Japan International Coordinating Agency Dominica Medical Information System Project (World Bank)

**Table 5:** Examples of adaptation options to reduce climate change risks to health in Dominica. Source: Reference [16].

**Near-Term Health Adaptation Options**
Strengthen solid waste management services across the island by increasing capacity of the DSWMC Enhance enforcement of existing legislation on waste management, vector control, and food safety Increase public awareness of health risks associated with climate change and encourage public involvement in adaptation efforts, with a particular focus on the engagement of unemployed youth Provide training to health sector staff on the health impacts of climate change and how to reduce risks Improve the reliability and safety of water storage practices at community and household levels Improve climate change and health data collection methods and enhance environmental monitoring Enhance the integration of climate services into health decision-making Strengthen the organizational structure of emergency response and ensure workers are familiar with emergency plans Ensure sufficient resources are available to efficiently respond to emergencies
**Longer-Term Health Adaptation Options**
Develop early warning systems for climate-sensitive health risks utilizing forecast information from the Caribbean Institute for Meteorology and Hydrology Continue studies on the impacts of climate change on vector-borne, waterborne, and foodborne diseases and on food insecurity, including analyses of weather, vector, and epidemiologic data Develop a national electronic database for health, vector, water, weather, and climate data that is accessible by all relevant stakeholders Conduct an assessment of data being collected by various departments and organizations to avoid duplication of efforts and promote collaboration Establish mechanisms for the routine flow of information (from weather, climate, vector, water, and disease surveillance systems) between key departments (e.g., Environmental Health Department, National Pest and Termite Control Company, Dominica Meteorological Services, Division of Agriculture, and DOWASCO) Conduct routine analyses of weather, vector, and epidemiologic data and distribute findings in a bulletin to relevant stakeholders Hold community meetings to increase awareness of protective behaviors through coordination among village councils, the Environmental Health Department, and health centers Convert current paper-based data collection systems to electronic systems Increase the capacity of laboratory facilities to detect disease-causing pathogens in humans, animals, and plants Increase the climate resiliency of health facilities and farm infrastructure Develop emergency plans and clarify departmental roles
